# Evaluation of CYP2C19 activity using microdosed oral omeprazole in humans

**DOI:** 10.1007/s00228-022-03304-3

**Published:** 2022-03-03

**Authors:** Annika Elbe, Kathrin Isabelle Foerster, Antje Blank, Peter Rose, Jürgen Burhenne, Walter Emil Haefeli, Gerd Mikus

**Affiliations:** Department of Clinical Pharmacology and Pharmacoepidemiology, Heidelberg University Hospital, Im Neuenheimer Feld 410, 69120 Heidelberg, Germany

**Keywords:** CYP2C19, Omeprazole, Inhibition, Induction, Microdose

## Abstract

**Purpose:**

To investigate the suitability of microdosed oral omeprazole for predicting CYP2C19 activity in vivo in combination with simultaneous assessment of CYP3A and CYP2D6 activity using both microdosed midazolam and yohimbine.

**Methods:**

An open, fixed-sequence study was carried out in 20 healthy participants. Single microdosed (100 µg) and therapeutic (20 mg) doses of omeprazole were evaluated without comedication and after administration of established CYP2C19 perpetrators fluconazole (inhibition) and rifampicin (induction). To prevent degradation of the uncoated omeprazole microdose, sodium bicarbonate buffer was administered. The pharmacokinetics of omeprazole and its 5-hydroxy-metabolite were assessed as well as the pharmacokinetics of midazolam and yohimbine to estimate CYP3A4 and CYP2D6 activity.

**Results:**

Calculated pharmacokinetic parameters after administration of 100 µg and 20 mg omeprazole in healthy subjects suggest dose proportionality. Omeprazole clearance was significantly decreased by fluconazole from 388 [95% CI: 266–565] to 47.2 [42.8–52.0] mL/min after 20 mg omeprazole and even further after 100 µg omeprazole (29.4 [24.5–35.1] mL/min). Rifampicin increased CYP2C19-mediated omeprazole metabolism. The omeprazole hydroxylation index was significantly related to omeprazole clearance for both doses. Both fluconazole and rifampicin altered CYP3A4 activity whereas no change of CYP2D6 activity was observed at all.

**Conclusions:**

Microdosed oral omeprazole is suitable to determine CYP2C19 activity, also during enzyme inhibition and induction. However, the administration of sodium bicarbonate buffer also had a small influence on all victim drugs used.

**Trial registration:**

EudraCT: 2017–004270-34.

**Supplementary information:**

The online version contains supplementary material available at 10.1007/s00228-022-03304-3.

## Introduction

Cytochrome P450 2C19 (CYP2C19) is an important enzyme of the cytochrome P450 enzyme family with large interindividual differences in the CYP2C19 activity due to genetic polymorphisms [1]. These differences in activity result in high interindividual variability of the metabolism of proton pump inhibitors (e.g., omeprazole), antiepileptic agents (e.g., brivaracetam, barbiturates, phenytoin), antiplatelet drugs (e.g., clopidogrel), and antidepressants (e.g., citalopram) [2–4]. Due to the individual allelic constellation, a population can be classified into 4 distinct CYP2C19 phenotypes, poor metabolizers (PM), intermediate metabolizers (IM), extensive metabolizers (EM), and ultrarapid metabolizers (UM) [5, 6]. Genetic differences also modify the propensity of the carrier for drug interactions and concurrent drug use can further modulate CYP2C19 activity resulting in a continuum of enzyme activities and potential discordances of phenotype and genotype [7]. Therefore, instead of the crude genetic classification, a more precise activity of the CYP2C19 isozyme can be determined by phenotyping with sensitive probe drugs.

Omeprazole as a commonly used proton pump inhibitor has previously been used to phenotype CYP2C19 activity using a therapeutic dose (20 or 40 mg single oral dose) [8]. A “hydroxylation index” is determined from a single 3 h blood sample, where the molar ratio of omeprazole to 5-OH-omeprazole plasma concentration is calculated [9]. This index reflects CYP2C19 activity and is related to the CYP2C19 genotype in the absence of interacting co-medication [10]. However, even a single oral dose of omeprazole can cause drug interactions by alteration of gastric pH (i.e., omeprazole reduces the AUC and C_max_ of the protease inhibitor nelfinavir by approximately one third [11]), or by competitive inhibition of CYP2C19 (decrease of AUC and C_max_ of active metabolite M8 of nelfinavir by 92% and 89%, respectively [11]). A microdose of omeprazole could avoid these interactions and could therefore be more suitable for CYP2C19 phenotyping because it does not cause autoinhibition of the target of interest (CYP2C19) and also does not cause gastrointestinal pH changes. Indeed, an exploratory study with some major limitations (small sample size, no CYP2C19 genotyping performed) was published in which 100 µg omeprazole was orally administered to explore the applicability of a microdose in a drug-drug interaction (DDI) trial [12]. However, microdosed intravenous omeprazole has been shown to represent hepatic CYP2C19 activity recently [13].

In this study, we investigated whether microdosed oral omeprazole is suitable to determine CYP2C19 activity in vivo in comparison to a therapeutic dose, also under conditions of CYP2C19 inhibition and induction*.* Since we have previously established midazolam and yohimbine microdosing to determine CYP3A and CYP2D6 activity [14–17], the additional simultaneous determination of CYP2C19 activity is a logical step to further develop a microdosed cocktail for phenotyping of the most important human CYP isozymes. Furthermore, the use of the hydroxylation index after the omeprazole microdose was evaluated in relation to the CYP2C19 genotype.

## Materials and methods

The study was approved by the Competent Authority (BfArM, Bonn) in Germany (EudraCT No: 2017–004270-34) and the responsible Ethics Committee of the Medical Faculty of Heidelberg University. It was conducted at the DIN EN ISO9001-certified Clinical Research Unit (KliPS) of the Department of Clinical Pharmacology and Pharmacoepidemiology in accordance with the standards of Good Clinical Practice (as defined in the ICH E6 Guideline for Good Clinical Practice) and in agreement with the Declaration of Helsinki and all specific legal requirements in Germany.

### Study population

All participants were mentally and physically healthy as confirmed by the medical history, a physical examination, a 12-lead electrocardiogram, and appropriate laboratory analyses. The participants had to undergo a urine drug screening and women were tested for pregnancy. Participants could be included in the trial if none of the following exclusion criteria were present: intake of any continuous medication other than oral contraceptives or any other substance including grapefruit juice known to interact with drug metabolizing enzymes or drug transporters, any condition, which could potentially modify absorption, distribution, metabolism, or excretion of the study drugs, allergies (except for mild forms of hay fever) or history of hypersensitivity reactions, smoking, excessive alcohol drinking, blood donation or participation in a clinical trial within the last month, positive drug screening or known or admitted drug abuse, and inability to communicate well with the investigator. Neither pregnant nor lactating women were included. All participants were required to consent to the use of two appropriate contraception methods and gave their written informed consent before any study measures were carried out.

All participants had a known CYP2C19 genotype previously determined in a genotyping study where common cytochrome isozyme polymorphisms were examined. CYP2C19 genotyping was performed for CYP2C19*2 (rs4244285), CYP2C19*3 (rs4986893), and for CYP2C19*17 (rs12248560) as previously described [13] and the presence of two wild type alleles was assumed if none of the tested polymorphisms was present.

### Study design and blood sampling

**Fig. 1:**
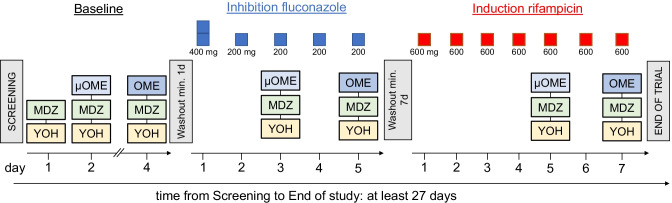
Study design of the clinical trial. MDZ = 10 µg midazolam; YOH = 50 µg yohimbine; µOME = 100 µg omeprazole solution; OME = 20 mg omeprazole capsule

This was an open-label clinical trial with fixed sequence design consisting of a total of seven inpatient study days and eight short visits for each participant. The study was conducted in 3 phases separated by 2 washout periods in which omeprazole microdose, omeprazole regular dose, CYP2C19 inhibition, and CYP2C19 induction were tested (Fig. 1).

Omeprazole was administered orally as a regular dose of 20 mg (OMEP® 20 mg, gastro-resistant capsule, HEXAL AG, Holzkirchen, Germany). The microdose of 100 µg omeprazole was freshly prepared using powder for solution for infusion (OMEP® 40 mg, powder for solution for infusion, HEXAL) that had been dissolved in 5 mL of NaCl (0.9% w/v); 12.5 µL (= 100 µg omeprazole) of this solution was diluted in 100 mL sodium bicarbonate (4.2% w/v). Ten minutes prior to omeprazole dosing, 50 mL sodium bicarbonate buffer (4.2% w/v) was administered to increase gastric pH to prevent degradation of the uncoated omeprazole.

Midazolam and yohimbine were used as a biomarker for CYP3A and CYP2D6 activity. Simultaneously with the omeprazole dose, each participant received a freshly prepared solution of 10 µg midazolam on every inpatient study day (Dormicum® V 5 mg/5 mL solution for injection, Roche, Grenzach-Wyhlen, Germany) and an oral dose of 50 µg yohimbine (Yohimbinum hydrochloricum D4®; Deutsche Homöopathie Union, Karlsruhe, Germany, containing 25 µg per tablet).

The CYP2C19 perpetrator fluconazole (Fluconazol HEXAL® 200 mg, HEXAL) was given for 5 days in oral doses of 400 mg on day 1 and 200 mg on days 2 to 5. After a washout period of at least 7 days, rifampicin was given for 7 days using a daily dose of 600 mg (Eremfat® 600 mg, RIEMSER Pharma GmbH, Greifswald, Germany). A diary was used to ensure correct intake of fluconazole and rifampicin at home.

On each pharmacokinetic study day, 16 blood samples of 4.9 mL each were collected to determine omeprazole, midazolam, and yohimbine concentrations. In the first study phase with midazolam and yohimbine, only 5 blood samples of 2.7 mL each were obtained (limited sampling pre-dose, 2, 2.5, 3, and 4 h after administration of study medication). In the 3 subsequent phases, samples were taken before pre-dose and 5, 10, 15, 20, 30, 45 min and 1, 1.5, 2, 2.5, 3, 4, 6, 8, and 24 h after administration of study medication. Blood samples were centrifuged for 10 min at 2500* g* and 4 °C, and separated plasma was distributed into 4 aliquots, each stored at − 20 °C until analysis.

### Quantification of omeprazole, 5-OH-omeprazole, midazolam, and yohimbine

Plasma concentrations of omeprazole and 5-OH-omeprazole after the 20 mg and 100 µg dose were analyzed with 2 different methods using high-performance and ultra-high performance liquid chromatography-tandem mass spectrometry (LC–MS/MS and UPLC-MS/MS) [13]. Calibration ranges were between 1–1000 ng/mL (LC–MS/MS) and 10–10,000 pg/mL (UPLC-MS/MS), respectively. The lower limits of quantification (LLOQ) for therapeutic doses were 1 ng/mL for omeprazole and 5-OH-omeprazole with within-batch and between-batch accuracies of 98–104% and precision of ≤ 7.0%. The LLOQ for the microdose were 10 pg/mL for both, omeprazole and 5-OH-omeprazole. Plasma concentrations could be quantified with within-batch and between-batch accuracies of 99–109% and precision of ≤ 13.2% [13].

Midazolam concentrations in plasma were determined by UPLC-MS/MS. The assay’s LLOQ was 0.37 pg/mL, and the accuracy/precision values were 96–99%/ ≤ 12.0%, respectively [18]. Yohimbine plasma concentrations were determined using an UPLC-MS/MS assay with an LLOQ of 5 pg/mL; accuracies ranged between 88 and 96% and precision was ≤ 11.7% [19].

### Data analysis

Standard non-compartmental pharmacokinetic parameters of omeprazole and 5-OH-omeprazole were calculated using Kinetica 5.0 (Thermo Fisher Scientific, Waltham, MA, USA): maximum plasma concentration (C_max_), time to reach C_max_ (t_max_), terminal elimination half-life (t_1/2_), area under the plasma concentration–time curve extrapolated to infinity (AUC_tot_), apparent volume of distribution at steady-state (V_ss_), apparent volume of distribution (V_z_), and apparent oral clearance (Cl/F). In addition, as a metric for CYP2C19 activity, the hydroxylation index (HI) was calculated as the molar ratio of the concentrations of omeprazole and 5-OH-omeprazole at 3 h [8].

For CYP3A activity evaluation, a limited sampling strategy based on midazolam AUC from 2 to 4 h was used to calculate the metabolic clearance as described previously [20]. To evaluate the activity of CYP2D6, the yohimbine clearance (Cl/F) was calculated by non-compartmental analysis using Kinetica 5.0 [17]. Data are presented as geometric mean and 95% confidence intervals (95% CI) unless stated otherwise.

To analyze the differences of pharmacokinetic parameters between microdosed and regular dosed omeprazole as well as the influence of CYP2C19 perpetrators, analysis of variance after logarithmic transformation and respective post hoc tests were used. A *p* value < 0.05 was considered significant. In addition, the geometric mean ratios and their respective 90% CIs have been calculated for C_max_, AUC and Cl/F with no interaction concluded if the 90% confidence interval includes 1. The statistical analysis was performed using Prism 8.4 (GraphPad Software Inc., La Jolla, CA, USA).

## Results

Twenty healthy non-smoking participants (15 females) were eligible for this clinical trial. They were between 20 and 48 years of age with an average of 29.4 ± 8.2 years. Seven were UM (CYP2C19*17/*17, *1/*17), 6 EM (*1/*1), 6 IM (*1/*2, *2/*17), and one was PM (*2/*2).

### Omeprazole

**Fig. 2:**
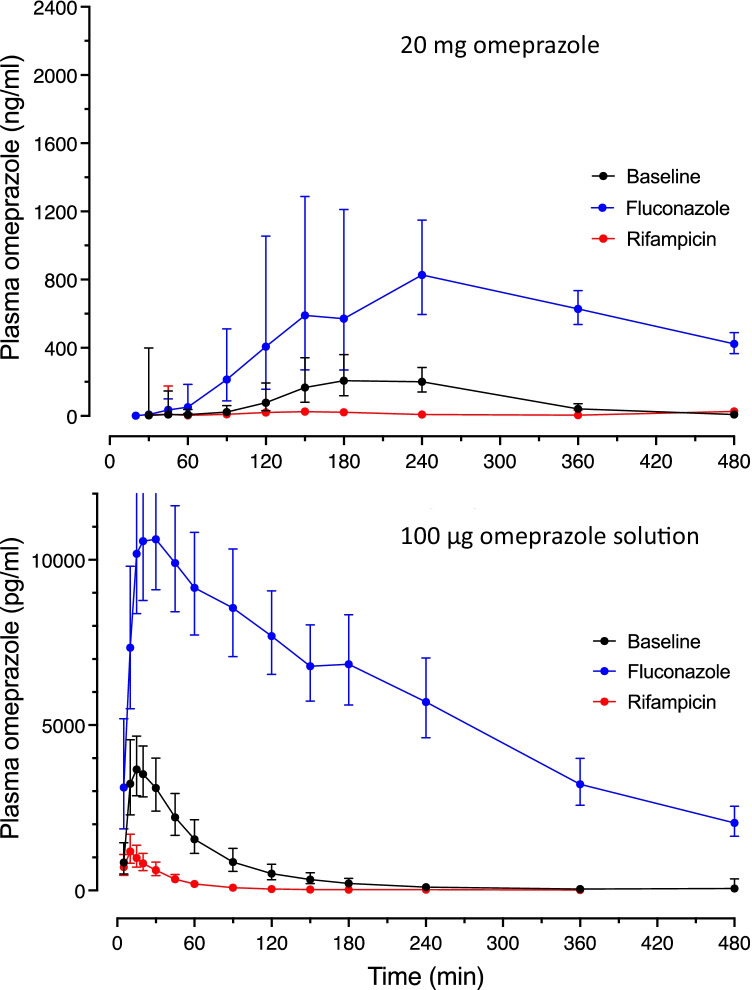
Geometric mean ± 95% CI plasma concentration vs. time curves for normal dosed omeprazole (upper panel) and microdosed omeprazole (bottom panel) during baseline condition (black) and during concomitant intake of fluconazole (blue) or rifampicin (red)

The plasma concentration–time profiles after a single oral dose of a 20 mg commercial capsule differed between the study conditions (Fig. 2, upper panel). Inhibition of CYP2C19 using fluconazole significantly increased C_max_ 3.25-fold with unchanged t_max_, induction of CYP2C19 by rifampicin significantly decreased C_max_ by 85% with unchanged t_max_ (Table 1). CYP2C19 induction and inhibition were reflected in all other pharmacokinetic parameters, with omeprazole clearance decreasing significantly by 88% during fluconazole and increasing 11-fold during rifampicin (Table 1). Compared to baseline, the AUC of the metabolite 5-OH-omeprazole was slightly decreased during inhibition and profoundly decreased during induction, while the terminal elimination half-life of the metabolite was prolonged during inhibition and reduced during induction (Table 1).

**Table 1:** Pharmacokinetics of omeprazole (20 mg orally) and its metabolite 5-OH-omeprazole alone (baseline), during CYP2C19 inhibition with fluconazole, and during induction with rifampicin in 20 healthy study participants

20 mg omeprazole	Baseline		Fluconazole		Rifampicin	
	Omeprazole		Omeprazole		Omeprazole	
Parameter [unit]	Geometric mean	95% CI	Geometric mean	95% CI	Geometric mean	95% CI
C_max_ [ng/mL]	373	255–545	1213 ^*^	1060–1389	56.6 ^*^	40.8–78.7
t_max_ [min] (harmonic mean)	178	159–204	169	143–207	143	120–178
AUC_tot_ [ng/mL min]	51,596	35,377–75,251	423,874 ^*^	384,463–467,324	4558 ^*^	3214–6463
t_1/2_ [min]	56.0	45.4–69.1	206 ^*^	189–225	27.2 ^*^	23.4–31.5
V_SS_ [L]	90.8	63.3–130	20.0 ^*^	17.6–22.7	715 ^*^	523–977
V_Z_ [L]	31.3	23.4–41.8	14.0 ^*^	12.7–15.6	172 ^*^	128–231
Cl/F [mL/min]	388	266–565	47.2 ^*^	42.8–52.0	4388 ^*^	3095–6222
	5-OH-omeprazole		5-OH-omeprazole		5-OH-Omeprazole	
Parameter [unit]	Geometric mean	95% CI	Geometric mean	95% CI	Geometric mean	95% CI
C_max_ [ng/mL]	136	107–174	41.1 ^*^	34.7–48.7	92.6 ^*^	76.2–113
AUC_tot_ [ng/mL min]	25,951	22,878–29,436	21,051 ^*^	18,851–23,507	9165 ^*^	7928–10,594
t_1/2_ [min]	83.6	58.8–119	277 ^*^	246–313	38.5 ^*^	32.4–45.8

Test baseline vs. fluconazole or rifampicin: *p < 0.05

*AUC*_*tot*_ area under the concentration–time curve, *Cl/F* apparent oral clearance, *C*_*max*_ maximum plasma concentration, *t*_*max*_ time to reach C_max_, *t*_*1/2*_ terminal elimination half-life, *V*_*SS*_ apparent volume of distribution at steady state, *V*_*Z*_ apparent volume of distribution associated with the terminal phase

The plasma concentration–time profiles after a single oral 100 µg dose of omeprazole as a solution differed substantially from the corresponding profiles after administration of the 20-mg omeprazole gastro-resistant capsule, which was also true for conditions of inhibition and induction of CYP2C19 (Fig. 2). C_max_ occurred much earlier after administration of the solution (t_max_ = 14.8 min) (Table 2) than after the commercial capsule (t_max_ = 178 min) (Table 1). As for the regular dose, all pharmacokinetic parameters of the microdose obtained during induction and inhibition significantly differed from the corresponding baseline values (Table 2). Induction and inhibition of CYP2C19 was reflected in omeprazole clearance, which was significantly decreased during fluconazole to 7.8% of baseline clearance and increased 5.8-fold during rifampicin (Table 2). The AUC of 5-OH-omeprazole was significantly decreased during inhibition and induction compared to baseline, while terminal elimination half-life was increased during inhibition and reduced during induction (Table 2). These effects are comparable to those observed after 20 mg omeprazole (Table 1).

**Table 2:** Pharmacokinetics of omeprazole (100 µg orally) and its metabolite 5-OH-omeprazole alone (baseline), during CYP2C19 inhibition with fluconazole, and during induction with rifampicin in 20 healthy study participants

100 µg omeprazole	Baseline		Fluconazole		Rifampicin	
	Omeprazole		Omeprazole		Omeprazole	
Parameter [unit]	Geometric mean	95% CI	Geometric mean	95% CI	Geometric mean	95% CI
C_max_ [ng/mL]	4.48	3.59–5.60	13.6 ^*^	11.5–16.1	1.31 ^*^	0.95–1.81
t_max_ [min] (harmonic mean)	14.8	12.5–18.2	21.8 ^*^	17.4–29.3	10.6 ^*^	17.4–29.3
AUC_tot_ [ng/mL min]	264	188–371	3405 ^*^	2845–4075	45.4 ^*^	33.0–62.4
t_1/2_ [min]	51.8	42.4–63.3	212 ^*^	200–224	36.0 ^*^	28.1–46.0
V_SS_ [L]	26.1	21.3–31.9	8.80 ^*^	7.50–10.3	93.1 ^*^	67.6–128
V_Z_ [L]	28.3	22.7–35.3	8.97 ^*^	7.54–10.7	114 ^*^	78.3–167
Cl/F [mL/min]	379	269–533	29.4 ^*^	24.5–35.1	2205 ^*^	1603–3034
	5-OH-omeprazole		5-OH-omeprazole		5-OH-omeprazole	
Parameter [unit]	Geometric mean	95% CI	Geometric mean	95% CI	Geometric mean	95% CI
C_max_ [ng/mL]	1.14	0.88–1.49	0.16 ^*^	0.13–0.19	0.97	0.84–1.13
AUC_tot_ [ng/mL min]	107	96.2–118	77.0 ^*^	66.4–89.2	46.9 ^*^	42.1–52.2
t_1/2_ [min]	70.4	61.1–81.0	239 ^*^	221–261	47.2 ^*^	43.4–51.4

Test baseline vs. fluconazole or rifampicin: *p < 0.05; **p < 0.01; ***p < 0.001; ****p < 0.0001

*AUC*_*tot*_ area under the concentration–time curve, *Cl/F* apparent oral clearance, *C*_*max*_ maximum plasma concentration, *t*_*max*_ time to reach C_max_, *t*_*1/2*_ terminal elimination half-life, *V*_*SS*_ apparent volume of distribution at steady state, *V*_*Z*_ apparent volume of distribution associated with the terminal phase

In Table 3, the geometric mean ratios of C_max_, AUC and the apparent oral omeprazole clearances are listed for the 2 doses and the 3 conditions. No dose-normalized AUC and clearance differences were observed between the omeprazole doses given alone indicating dose proportionality. A significant influence of both perpetrators (fluconazole and rifampicin) on omeprazole clearance was observed for both doses used but during either perpetrator drug omeprazole clearance after 100 µg was almost twofold lower than after 20 mg (Table 3).

**Table 3:** Geometric mean ratios (GMR) and 90% confidence intervals (90% CI) of dose-normalized omeprazole C_max_, AUC and apparent oral clearance (Cl/F) after 100 µg p.o. and 20 mg p.o. during baseline, CYP2C19 inhibition with fluconazole, and during induction with rifampicin in 20 healthy study participants

Cl/F (reference vs. test)		GMR	90% CI
20 mg vs. 100 µg omeprazole	C_max_ AUC Cl/F	2.4033 1.0235 0.9771	1.7620–3.2781 0.8706–1.2033 0.8311–1.1487
20 mg vs. 100 µg omeprazole during fluconazole	C_max_ AUC Cl/F	2.2383 1.6066 0.6223	1.9007–2.6359 1.3782–1.8728 0.5339–0.7253
20 mg vs. 100 µg omeprazole during rifampicin	C_max_ AUC Cl/F	4.6207 1.9899 0.5025	3.8107–5.6029 1.6648–2.3785 0.4204–0.6007
20 mg omeprazole at baseline vs. fluconazole	C_max_ AUC Cl/F	3.2556 8.2153 0.1217	2.4141–4.3905 6.1514–10.9713 0.0911–0.1626
20 mg omeprazole at baseline vs. rifampicin	C_max_ AUC Cl/F	0.1519 0.0883 11.32	0.1115–0.2069 0.0715–0.1091 9.17–13.98
100 µg omeprazole at baseline vs. fluconazole	C_max_ AUC Cl/F	3.0321 12.986 0.0775	2.4115–3.8124 9.560–17.395 0.0575–0.1046
100 µg omeprazole at baseline vs. rifampicin	C_max_ AUC Cl/F	0.2920 0.1717 5.82	0.2402–0.3550 0.1411–0.2091 4.78–7.09

### Hydroxylation index (HI)

**Fig. 3:**
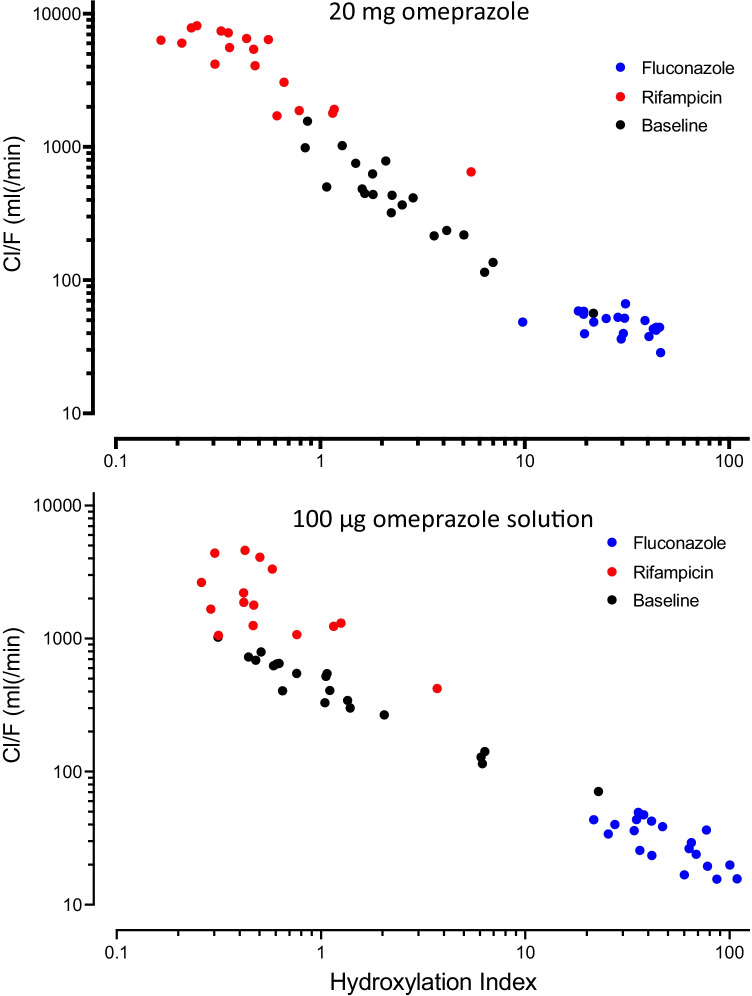
Relationship between omeprazole hydroxylation index and the omeprazole apparent oral clearance for normal dosed omeprazole (upper panel) and microdosed omeprazole (bottom panel) during baseline condition (black) and during concomitant intake of fluconazole (blue) or rifampicin (red). During induction with rifampicin in 5 participants, omeprazole was below LLOQ at 3 h after microdosed omeprazole; no hydroxylation index was calculated. This also occurred in 2 participants after normal dosed omeprazole

The hydroxylation index of omeprazole as the molar ratio of omeprazole to 5-OH-omeprazole plasma concentration from the single 3 h blood sample varied considerably by a factor of 278 (range: 0.1660–46.22) after 20 mg and by 419 (range: 0.2600–108.9) after 100 µg omeprazole (alone and during fluconazole and rifampicin) (Table 4). For both omeprazole doses used, the relationship between the HI and the apparent oral clearance is shown in Fig. 3. For both doses a log–log regression revealed an adjusted *r*^2^ > 0.9 and a slope close to − 1 (20 mg omeprazole: − 1.034; 100 µg omeprazole: − 0.8061). As the regression analysis mixes intra- and interindividual variability, the regression lines are not shown and the results are for illustration purposes only.

**Table 4:** Geometric mean and 95% confidence intervals (95% CI) of the omeprazole hydroxylation index during baseline, CYP2C19 inhibition with fluconazole, and during induction with rifampicin in 20 healthy study participants

	100 µg omeprazole		20 mg omeprazole	
	Geometric mean	95% CI	Geometric mean	95% CI
Baseline				
All (*n* = 20)	1.26	0.75–2.12	2.47	1.71–3.57
UM (*n* = 7)	0.92	0.35–2.41	2.0	1.04–3.84
EM (*n* = 6)	1.20	0.48–3.0	2.34	1.16–4.7
IM (*n* = 6)	1.18	0.46–3.0	2.31	1.28–4.18
PM (*n* = 1)	22.8		21.7	
Fluconazole				
All (*n* = 20)	49.3*	39.6–61.4	28.7*	23.5–35.0
UM (*n* = 7)	41.0	25.2–66.9	21.8	14.3–33.4
EM (*n* = 6)	58.4	34.9–97.6	29.8	20.6–43.3
IM (*n* = 6)	49.2	32.1–75.3	36.6	27.9–48.0
PM (*n* = 1)	65.1		44.0	
Rifampicin				
All (*n* = 20)	0.55	0.37–0.82	0.50*	0.33–0.74
UM (*n* = 7)	0.41	0.19–0.87	0.32	0.20–0.51
EM (*n* = 6)	0.51	0.34–0.79	0.55	0.28–1.08
IM (*n* = 6)	0.54	0.29–1.04	0.47	0.25–0.89
PM (*n* = 1)	3.70		5.46	

*Test all participants baseline vs. fluconazole or rifampicin: *p* < 0.05

### Midazolam

**Fig. 4:**
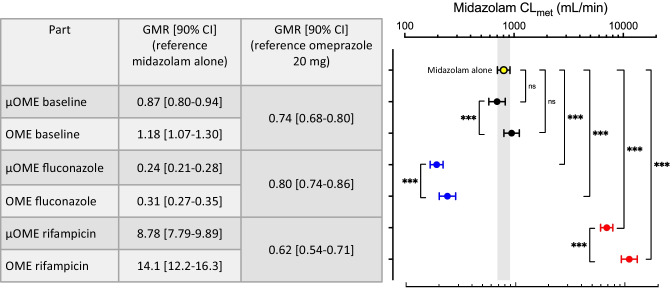
Calculated metabolic clearance of midazolam (geometric mean ± 95% confidence interval) in relation to the different study conditions (baseline condition (black), concomitant intake of fluconazole (blue), rifampicin (red)). The results of the repeated measures ANOVA after logarithmic transformation with Sidak’s multiple comparisons test. In addition, the geometric mean ratios (GMR) with their 90% confidence intervals (90% CI) are listed. ns = not significant; µOME = 100 µg omeprazole solution; OME = 20 mg omeprazole capsule. ***p < 0.005

The calculated metabolic clearance of midazolam as a marker of CYP3A activity during CYP2C19 inhibition and induction conditions was analyzed using repeated measures ANOVA and post hoc testing of every condition against baseline and between omeprazole microdose and normal dose. Compared to baseline without omeprazole, midazolam CL_met_ was unchanged irrespective of the administered omeprazole dose although the geometric mean ratios are different from 1 (Fig. 4). However, because both fluconazole and rifampicin also affect CYP3A activity, this resulted in a significant reduction of CL_met_ during fluconazole and significant increase of CL_met_ during rifampicin (Fig. 4). Comparison of midazolam CL_met_ between microdose and normal dose of omeprazole showed significantly lower clearances after omeprazole microdosing, irrespective of the condition (inhibition or induction) (Fig. 4).

### Yohimbine

Fluconazole and rifampicin did not modify yohimbine pharmacokinetics a marker of CYP2D6 activity (suppl Table 1). However, apparent yohimbine clearance was significantly lower after omeprazole microdoses than after regular doses both at baseline (2528 mL/min vs. 3274 mL/min) and during fluconazole (2224 mL/min vs. 2884 mL/min). Also, during rifampicin, yohimbine clearance was lower with microdosed omeprazole than with normal dose omeprazole but this difference did not reach significance (*p* = 0.0506; suppl Table 1).

### Safety and tolerability

All study drugs were well tolerated and no serious adverse events occurred. Most adverse events occurred after intake of fluconazole and rifampicin (mainly headaches and nausea), all were mild and transient, and no actions had to be taken.

## Discussion

### Microdose vs. therapeutic dose

We evaluated the suitability of the pharmacokinetics an oral omeprazole microdose (100 µg) to predict changes in CYP2C19 activity and compared it with the pharmacokinetics of a therapeutic dose (20 mg). Under baseline conditions, there were no significant differences of dose-normalized AUC, elimination half-life, and clearance between microdosed and therapeutic dosed omeprazole suggesting dose proportionality, which offers the possibility of replacing the therapeutic omeprazole dose by a microdose as phenotyping measure. However, significant differences regarding dose-normalized C_max_ and t_max_ were observed, which can be caused by the different dosage forms. Commercial gastro-resistant capsules (20 mg) were used with delayed release (t_max_ 178 min with C_max_ 327.7 ng/mL). For the omeprazole microdose, we prepared an oral solution with 75 mmol sodium bicarbonate and omeprazole to prevent the acid labile omeprazole from being destroyed in the stomach prior to absorption. In contrast, absorption of this omeprazole solution was fast (t_max_ 15 min) with correspondingly higher dose-normalized C_max_ (896 ng/mL). The amount of sodium bicarbonate administered with omeprazole seems to be important for absorption. Park and co-workers administered 10 mmol of sodium bicarbonate resulting in a relative bioavailability (F_rel_) of 0.35 of the microdose [13]. Bioequivalence studies showed that uncoated omeprazole in combination with 48 mmol sodium bicarbonate was bioequivalent to an encapsulated formulation [21]. In our study, 75 mmol sodium bicarbonate resulted in F_rel_ of 1.02, indicating successful prevention of acid destruction of the microdosed omeprazole solution.

### Omeprazole microdose vs. therapeutic dose during fluconazole

Fluconazole, a strong CYP2C19 inhibitor, increased C_max_ and AUC of the 20 mg omeprazole dose threefold and eightfold and reduced clearance to 12% of the baseline value. This confirms the already known strong inhibition of fluconazole [12]. Using 100 µg omeprazole, an even more pronounced inhibitory effect on CYP2C19 was observed with AUC increasing 12-fold and the clearance decreasing to 7.8% of baseline. Omeprazole has been reported to have non-linear pharmacokinetics after a single high dose and also repetitive administration due to autoinhibition of CYP2C19 [22, 23]. When using an omeprazole microdose, this autoinhibition disappears and the effect of an inhibitor is more pronounced. Although a time-dependent inhibitory effect of fluconazole has not been investigated in a clinical trial, it seems likely that there is no further increase of inhibition with time, since on day 5 of fluconazole, there was less inhibition (normal omeprazole dose) than on day 3 with the omeprazole microdose.

Because fluconazole is a competitive CYP2C19 inhibitor [24], higher doses and thereby higher concentrations of fluconazole will cause stronger inhibition. After a low dose of fluconazole (8 days of 50 mg fluconazole daily), a 2.7–2.8-fold increase of omeprazole AUC was found [12]. Doubling the fluconazole dose (100 mg fluconazole daily for 5 days) showed a sixfold increase of omeprazole AUC in healthy volunteers [25]. In our study, the dose regimen for fluconazole was according to the Summary of Product Characteristics [26]. With oral administration of 400 mg fluconazole on day 1 and 200 mg fluconazole on day 2 to 5, eightfold (20 mg omeprazole) and 12-fold (100 µg omeprazole) increases of omeprazole AUC were reached. A pH-mediated influence of omeprazole or sodium bicarbonate on fluconazole pharmacokinetics is unlikely since changes in gastric pH did not affect fluconazole C_max_, t_max_, and AUC [27].

### Microdose vs. therapeutic dose during rifampicin

Rifampicin is well characterized as an inducer of multiple cytochrome P450 isozymes, among them also CYP2C19 [28]. As expected, rifampicin increased omeprazole clearance irrespective of the omeprazole dose. However, when a microdose was used, omeprazole clearance increased 5.8-fold, whereas omeprazole clearance increased 11-fold after 20 mg omeprazole. CYP2C19 induction by rifampicin is mediated by an activation of CYP2C19 transcription, which is a time-dependent process. In our study, we administered rifampicin 600 mg daily for 7 days. It has been reported that maximal induction of intestinal and hepatic CYP3A4 was achieved in > 90% of participants within 5 and 10 days [29]. Other studies found that the maximum effect of rifampicin induction on CYP2C and CYP3A4 was achieved after 7 to 11 days [28, 30, 31]. Because the microdose of omeprazole was administered after 5 days of rifampicin and the 20 mg dose after 7 days, this might be the reason for the observed difference in the magnitude of induction. This is in line with the induction of the CYP3A4 substrate midazolam with an increase of metabolic clearance by 10.1-fold on day 5 and 11.9-fold on day 7 (Fig. 4), suggesting that induction was still evolving. The inducing effect of a low dose rifampicin (150 mg) on omeprazole pharmacokinetics decreased omeprazole AUC to 22% after a microdose and 20% after a 20 mg dose after 8 and 9 days of rifampicin treatment, respectively [12]. There was no significant difference between the 2 omeprazole doses, probably due to the long rifampicin treatment. Likely due to the lower rifampicin dose, the induction was not as pronounced compared to the observed AUC reduction to 17% (day 5, microdose) and 8% (day 7, 20 mg dose). In another study investigating the influence of rifampicin (6 days, 450 mg daily) on 20 mg omeprazole, omeprazole AUC decreased to 12.9% [32]. Comparing these three studies, a dose-dependent and time-dependent effect of rifampicin induction on omeprazole elimination is observed confirming the dependency of rifampicin-induced induction reported earlier [33].

Plasma concentrations of the metabolite 5-OH-omeprazole were also reduced during induction. Increased metabolite exposure would result during induction as the formation rate increases; however, further metabolism of 5-OH-omeprazole is partly catalyzed by CYP3A4 to omeprazole hydroxysulfone [34]. Therefore, with CYP3A4 also induced by rifampicin the exposure of 5-OH-omeprazole can be reduced due to enhanced secondary metabolism, which is also reflected in the decreased metabolite half-life.

### CYP2C19 genotype

Because the aim of this investigation was to test whether microdosed oral omeprazole can also be used to assess CYP2C19 activity in vivo, the CYP2C19 genotypes of the study participants are also important. Therefore, study participants were divided into four groups according to their CYP2C19 genotype: UM, EM, IM, and PM. No significant differences of omeprazole (20 mg) pharmacokinetics have been observed between UM, IM, and EM under baseline conditions as well as during fluconazole and also rifampicin. The same resulted using microdosed omeprazole (suppl. Figure 1).

In ultrarapid metabolizers with one or two CYP2C19*17 alleles CYP2C19 activity is increased and homozygous carriers of CYP2C19*17 had significantly lower exposure with the CYP2C19 substrates omeprazole and escitalopram than carriers of the wild-type allele [35, 36]. In our study, omeprazole clearance after 20 mg was 515 mL/min (95% CI: 260–1021 mL/min; *n* = 7) in ultrarapid metabolizers and 367 mL/min (95% CI: 188–720 mL/min; *n* = 6) in extensive metabolizers, which was significantly different. Similar clearances were observed after 100 µg omeprazole with 476 mL/min (95% CI: 238–952 mL/min) in UMs and 367 mL/min (95% CI: 194–693 mL/min) in EMs. However, the clearances showed a large overlap between the CYP2C19 genotypes (suppl. Figure 1). Only the single poor metabolizer of this study was easily detected under baseline conditions since threefold and 6.5-fold higher C_max_ and AUC were observed (after 20 mg omeprazole in comparison with the EM group) with omeprazole clearance being as low as 57 mL/min.

During fluconazole, omeprazole clearance was reduced to 49 mL/min (UM), 45 mL/min (EM), 47 mL/min (IM), and 44 mL/min (PM), respectively (suppl. Figure 1), confirming that fluconazole is a potent CYP2C19 inhibitor. Fluconazole is listed as a moderate CYP3A4 inhibitor by the FDA [37], which is confirmed by our data using midazolam. Since CYP3A4 plays a minor role in omeprazole metabolism, which can be confirmed by the small clearance reduction in the PM by fluconazole, the observed clearance reduction in CYP2C19 metabolizers can be attributed to CYP2C19 inhibition.

Enzyme induction using rifampicin profoundly increased omeprazole clearance to 6321 mL/min (UM), 3527 mL/min (EM), 4903 mL/min (IM), and 649 mL/min (PM) (suppl. Figure 1). This large difference between the PM and the other genotypes demonstrates the induction of CYP2C19. A more than tenfold increase of omeprazole clearance is observed in the one PM participant, which suggests induction of CYP3A and confirms previously observed data [32]. Similar results for 100 µg omeprazole were observed during fluconazole as well as rifampicin.

### Hydroxylation index (HI)

The omeprazole hydroxylation index (HI) has previously been used as marker for CYP2C19 phenotyping [8–10, 38]. After calculation of the HI, however, there were no significant differences between the CYP2C19 genotypes (Table 4). Only the one PM was clearly distinguished. Probably due to the low sample size of each genotype our results are in contrast to published data where 160 participants (113 EM, 40 IM, and 7 PM) [8] and 300 participants (124 EM, 129 IM, and 47 PM) [38] were studied. Since the HI is calculated from a single blood sample collected 3 h after omeprazole dosing, we observed differences between the 100 µg and 20 mg dose due to the different formulation with different t_max_ values.

### Midazolam

Microdosed midazolam as a CYP3A4 marker [14] in combination with a limited sampling methodology [20, 39] was able to assess the influence of inhibition and induction of CYP3A4 by fluconazole and rifampicin in this study. The metabolic clearance of midazolam during fluconazole decreased to 28% and 26% of the baseline value with 100 µg and 20 mg omeprazole, respectively. This corresponds well to the reported 3.6-fold increase of midazolam AUC after oral administration of 7.5 mg midazolam during fluconazole treatment [40]. Rifampicin as a non-selective inducer of multiple CYP enzymes increased the metabolic clearance of midazolam 10–12-fold. This is in accordance with the midazolam AUC decrease to 4.1% of the baseline value during treatment with 600 mg rifampicin daily over 5 days [41].

Because the effect of the perpetrators was studied with the 100 µg and 20 mg omeprazole dose, the design was suitable to also study the influence of omeprazole dose. The metabolic clearance of midazolam after 20 mg omeprazole was significantly higher than after administration of 100 µg omeprazole during baseline, fluconazole, and rifampicin. An auto-inhibitory effect of 20 mg omeprazole can be ruled out since this would have been the opposite effect, especially during baseline. Because the omeprazole microdose was always combined with sodium bicarbonate, an enhanced absorption of midazolam might have occurred [42, 43]. Midazolam alters its structure in solutions of pH > 6 and gets more lipophilic thus improving membrane permeability [44, 45].

### Yohimbine

Although there was no effect on CYP2D6 expected by the substances used in this study, we included microdosed yohimbine as a marker substance mainly to generate more data on a potential microdosed CYP cocktail. Both fluconazole and rifampicin as perpetrators did not alter the clearance of yohimbine. Decreased yohimbine clearance was observed when 100 µg omeprazole was administered instead of 20 mg, which was significant during baseline and fluconazole, but did not reach significance during rifampicin (suppl. Table 1). This is quite similar to the observations with midazolam, which likewise might be caused by the administration of sodium bicarbonate resulting in the same clearance alteration. So far, there are no data published on yohimbine regarding pH-dependent alteration of gastrointestinal absorption.

### Limitations

There are two major limitations in this study, one being the small sample size of the different CYP2C19 genotypes and the second the effect of the use of sodium bicarbonate in order to stabilize the liquid omeprazole microdose. We have included all known genotypes of CYP2C19, which actually resulted in a large clearance variability (> 14-fold) of omeprazole. The use of sodium bicarbonate altered yohimbine and midazolam pharmacokinetics when combining them with microdosed omeprazole for a microdosed CYP cocktail. This limits the usability and comparability of the cocktail approach with microdosed omeprazole. A gastro-resistant formulation of microdosed omeprazole (e.g., a capsule) might be necessary to eliminate the influence of the sodium bicarbonate solution.

In summary, this clinical study assessed the CYP2C19 activity using microdosed oral omeprazole in humans in comparison to a therapeutic 20 mg dose. Omeprazole microdoses appear similarly suitable for phenotyping of CYP2C19 as therapeutic doses. The extent of drug interactions with CYP2C19 inhibitors and inducers are in a similar order of magnitude as after therapeutic doses. The use of a microdosed CYP cocktail (omeprazole, midazolam, and yohimbine) was only slightly impacted by co-administering sodium bicarbonate to prevent disintegration of omeprazole in the peptic milieu but needs further investigation.

## Supplementary information

Below is the link to the electronic supplementary material.

Supplementary file1 (DOCX 146 KB)

## Data Availability

The datasets generated during and/or analyzed during the current study are available from the corresponding author on reasonable request.
