# Supplementary material for: Evaluation of CYP2C19 activity using microdosed oral omeprazole in humans
Source: Eur J Clin Pharmacol. 2022 Mar 3;78(6):975–87. doi: 10.1007/s00228-022-03304-3 (PMC9107402; doi:10.1007/s00228-022-03304-3)
Supplement: Supplementary file 1 — Supplementary file1 (DOCX 146 KB) [file 228_2022_3304_MOESM1_ESM.docx]

**Supplementary data**

Evaluation of CYP2C19 activity using microdosed oral omeprazole in humans

Annika Elbe, Kathrin Isabelle Foerster, Antje Blank, Peter Rose, Jürgen Burhenne, Walter Emil Haefeli, Gerd Mikus

**Supplementary table 1:** Geometric mean and 95 % confidence intervals (95 % CI) of yohimbine pharmacokinetic parameters during baseline, CYP2C19 inhibition with fluconazole, and during induction with rifampicin in 20 healthy study participants.

| Baseline | 100 µg omeprazole | | 20 mg omeprazole | |
| --- | --- | --- | --- | --- |
| Parameter [unit] | Geometric mean | 95 % CI | Geometric mean | 95 % CI |
| C_max_ [pg/ml] | 350^x^ | 198-619 | 217^x^ | 118-398 |
| AUC_tot_ [pg*ml/h] | 330^x^ | 150-725 | 255^x^ | 114-566 |
| t_1/2_ [min] | 64.0 | 45.6-89.9 | 64.2 | 46.3-89.0 |
| Cl/F [ml/min] | 2528^x^ | 1150-5560 | 3274^x^ | 1472-7283 |
| **Fluconazole** | **100 µg omeprazole** | | **20 mg omeprazole** | |
| Parameter [unit] | Geometric mean | 95 % CI | Geometric mean | 95 % CI |
| C_max_ [pg/ml] | 427^x, #^ | 254-718 | 288^x^ | 159-522 |
| AUC_tot_ [pg*ml/h] | 375^x^ | 167-842 | 289^x^ | 124-676 |
| t_1/2_ [min] | 64.3 | 45.4-91.3 | 54.6 | 37.7-79.1 |
| Cl/F [ml/min] | 2224^x^ | 990-4997 | 2884^x^ | 1233-6749 |
| **Rifampicin** | **100 µg omeprazole** | | **20 mg omeprazole** | |
| Parameter [unit] | Geometric mean | 95 % CI | Geometric mean | 95 % CI |
| C_max_ [pg/ml] | 267^#^ | 158-449 | 188 | 110-320 |
| AUC_tot_ [pg*ml/h] | 244 | 122-489 | 182 | 88.7-373 |
| t_1/2_ [min] | 56.4 | 42.9-74.1 | 58.4 | 45.7-74.6 |
| Cl/F [ml/min] | 3420 | 1705-6859 | 4584 | 2236-9396 |
| AUC_tot_: area under the concentration-time curve; t_1/2:_ terminal elimination half-life; Cl/F: apparent oral clearance, C_max_: maximum plasma concentration.  Test microdosed vs. normal dosed omeprazole (same comedication) p < 0.05: ^x^  Test perpetrator vs. baseline (same omeprazole dose) p < 0.05: ^#^  Fluconazole: 400 mg on day 1, 200 mg on day 2-4,  Rifampicin: 7 days 600 mg rifampicin qd. | | | | |

**Supplementary figure 1:** Omeprazole clearance after 100 µg and 20 mg doses in realtion to the CYP2C19 genotype during baseline condition (upper plot), during concomitant intake of fluconazole (middle plot), and rifampicin (lower plot).
